# Correlation of *HER2*, *MDM2*, *c-MYC*, *c-MET*, and *TP53* Copy Number Alterations in Circulating Tumor Cells with Tissue in Gastric Cancer Patients: A Pilot Study

**DOI:** 10.29252/ibj.24.1.47

**Published:** 2019-08-28

**Authors:** Fatemeh Nevisi, Marjan Yaghmaie, Hossein Pashaiefar, Kamran Alimoghaddam, Masoud Iravani, Gholamreza Javadi, Ardeshir Ghavamzadeh

**Affiliations:** 1 Department of Biology, Science and Research Branch, Islamic Azad University, Tehran, Iran;; 2 Hematology, Oncology and Stem cell Transplantation Research Center, Tehran University of Medical Sciences, Tehran, Iran;; 3 Department of Medical Genetics, School of Medicine, Tehran University of Medical Sciences, Tehran, Iran;; 4 Masood GI and Hepatology Clinic, Tehran, Iran

**Keywords:** Circulating tumor cells, Fluorescence *in situ* hybridization, Gene amplification

## Abstract

**Background:**

The analysis of the gene copy number alterations in tumor samples are increasingly used for diagnostic and prognostic purposes in patients with GC. However, these procedures are not always applicable due to their invasive nature. In this study, we have analyzed the copy number alterations of five genes (*HER2*, *MDM2*, *c-MYC*, *c-MET*, and *TP53*) with a fixed relevance for GC in the CTC of GC patients, and, accordingly, as a potential approach, evaluated their usage to complete primary tumor biopsy.

**Methods:**

We analyzed the status of the copy number alterations of the selected genes in CTC and matched biopsy tissues from 37 GC patients using FISH.

**Results:**

*HER2* amplification was observed in 2 (5.41%) samples. *HER2* gene status in CTC showed a strong agreement with its status in 36 out of 37 patients’ matched tissue samples (correlation: 97.29%; Kappa: 0.65; *p* < 0.001). *MDM2* amplification was found only in 1 (2.70%) sample; however, the amplification of this gene was not detectable in the CTC isolated from this patient. *c-MYC* amplification was observed in 3 (8.11%) samples, and the status of its amplification in the CTC indicated a complete agreement with its status in the matched tissue samples (correlation: 100%; Kappa: 1.0).

**Conclusion:**

Our work suggests that the amplification of *HER2* and *c-MYC* is in concordance with the CTC and achieved biopsies, and, consequently, CTC may act as a non-invasive alternative for recording the amplification of these genes among GC patients.

## Introduction

Gastric cancer is the fourth most common cancer with high morbidity and mortality; it leads to more than 70,000 deaths annually worldwide^[^^1^^,^^2^^]^. Despite the steady decline in the incidence of the mortality, there are some limitations for diagnostic and therapeutic process of the GC treatment that include the lack of precise diagnostic tests for the early detection and the absence of valuable prognostic factors. Therefore, for improving the management of GC patients, attention should be focused on the development of appropriate diagnostic and monitoring tools. During the past recent decades, numerous studies have shown the potential efficiency of CTC, as a novel blood-based biomarker for diagnostic, prognostic and therapeutic purposes for various types of cancers, including GC^[^^3^^]^. 

Historically, the major source of material for the evaluation of the genetic changes of tumor cells are the tumor tissues obtained from surgical or biopsy specimens. However, due to several limitations, such tumor tissue-based strategies cannot be carried out routinely. It may even be impossible to obtain a tissue specimen, especially in metastatic cases with anatomical challenges. Likewise, the tissues obtained from biopsy may not always be adequate for the detection of genetic alterations occurring in tumor cells. Besides, due to the genomic difference between the primary and metastatic tumors arising from the same patient, the tumor tissue acquired at the time of diagnosis may not reflect the genetic alterations observed at the time of clinical progression. Moreover, chemotherapy or targeted therapy may lead to genetic variation in the tumor cells. In such cases, CTC may be a helpful approach for detecting the current status of tumor features, allowing repeated samplings and providing useful and timely information for determining the best treatment options. Furthermore, detecting CTC and determining the genetic changes in these cells may result in a better and real-time understanding of the tumor genetic profile. Studies have shown that the presence of CTC and the changes in their counts in the blood stream of GC patients may be a useful tool for predicting the prognosis of these patients and the progression of the tumor, and, therefore, may act as a good monitoring marker for chemotherapy^[^^4^^,^^5^^]^. 

The molecular characterization of CTC is complex because of the relatively low number of CTC and their dilution by non-tumor cells. FISH is an effective method for evaluating the gene copy number in the individual cells of a heterogeneous cell population^[^^6^^]^ and has been successfully applied for tumor cells isolated from the blood^[^^7^^]^. Therefore, in this study, using FISH, we analyzed the copy number alterations of five genes (*HER2*, *MDM2*, *c-MYC*, *c-MET*, and *TP53*) in the CTC obtained from GC patients to evaluate their usage as a potential complement approach for primary tumor biopsies. The objective of this study was to determine whether the molecular study of CTC can act as an alternative source of tissue specimens to GC patients.

## MATERIALS AND METHODS

**Patients**

Peripheral blood samples were obtained from 37 patients with metastatic GC who were treated at the Madaen and Sina Hospitals, and Masoud Clinic (Tehran, Iran). All patients received intravenously 60 mg/m^2^ of Taxotere and 60 mg/m^2^ of Oxaliplatin, followed by continuous IV infusion of 5-fluorouracil 5-FU (500 mg/m^2^) over 24 hours. Formalin-fixed paraffin-embedded tissues from the primary tumors were obtained from the pathology core at the Madaen and Sina Hospitals. The pathological staging of the disease was performed based on the revised tumor-node-metastasis classification system^[^^8^^]^. The histological diagnosis was based on the World Health Organization criteria^[^^9^^]^. An informed consent was obtained from each patient, and the study was approved by the Ethics Committee (TUMS-30076) of Tehran University of Medical Sciences, Tehran, Iran.

**Isolation of CTC**

The peripheral blood samples were collected from the GC patients and transferred into a 10-mL CellSave Preservative Tube (Veridex, Raritan, NJ, USA). Peripheral blood mononuclear cells were immediately isolated by Ficoll-Paque (GE Healthcare, Waukesha, WI, USA) density-gradient centrifugation, according to the manufacturer’s instructions. Phosphate-buffered saline was used to wash the cell pellets (0.15 M, pH 7.4). CTC were isolated from the peripheral blood mononuclear cell using the Dynabeads CD45 kit (Invitrogen, Carlsbad, CA) according to the manufacturer’s instructions based a negative selection methodology. Accordingly, CD45^-^ cells with intact 4′,6-diamidino-2-phenylindole nuclei exhibiting tumor-associated morphologies were classified as CTC.

**FISH on paraffin-embedded tissue sections**

The paraffin-embedded tissues were cut into 3-5-micron sections using a microtome. One H & E stained slide from each patient was examined by an expert pathologist to mark the malignant cell areas. The sections were placed on positive-charged slides (Menzel-Gläster, Braunschweig, Germany), deparaffinized and rehydrated through an ethanol series, which were subsequently air-dried. To identify *HER2*, *MDM2*, *c*-MYC, *c*-MET, and P53 copy number alterations, FISH was performed as previously described^[^^10^^]^. The probes *HER2*/CE17, *MDM2*/CE12, and *TP53*/NF1 (Cytocell, UK) as well as *c-MET*/CE7 and *c-MYC*/CE8 (Metasystems, Germany) were applied on malignant cells. The hybridized slides were examined under an Olympus BX51 microscope (Olympus, Tokyo, Japan) at 100 magnification. *MDM2*, *c-MYC*, and *c-MET* were considered as amplified copies if *MDM2*/CEP12, c-*MYC*/CEP8, and c-*MET*/CEP7 ratio were of ≥2, or more than four of the copies of these genes in ≥40% of the analyzed cells were observed (high polysomy)^[^^11^^]^. The cut-off value of 5% was used for the evaluation of the *TP53* deletion. 

**FISH on CTC**

Following CTC enrichment, the slides were prepared and immersed in 2 sodium chloride-sodium citrate buffer at room temperature for 2 minutes. The slides were then dehydrated in a graded series of ethanol solutions (70, 85, 90, and 100%, each for 1-2 minutes). Afterward, 1.5 μl of the probe mixture was added to the cells on the slides and covered with a coverslip. Denaturation was performed at 76 °C for 5 minutes, and hybridization was carried out at 37 °C for 18 hours. Following the hybridization, the slides were washed in 0.4 sodium chloride-sodium citrate at 72 °C for 2 minutes and then were immersed in 2 sodium chloride-sodium citrate /0.1% NP-40 (pH 7.0) solution at room temperature for one minute. After loading 10 μl of 4′,6-diamidino-2-phenylindole (Cytocell, UK) onto each slide, using a fluorescence microscope, FISH signals were evaluated on a minimum of 100 interphase nuclei with prominent nucleoli (Olympus, BX50, Japan). The same cut-off values of the tissues were used to evaluate the copy number alterations of the selected genes in the CTC.

**Statistical Analysis**

Descriptive statistical analyses, including Cohen’s kappa testing, were performed using the SPSS 20.0 software package (SPSS, Chicago, IL, USA). Specificity and sensitivity for *HER2* and *c-MYC* amplification detection in CTC were calculated based on the classification of patients for *HER2* and *c-MYC* amplification status achieved from the archival tissues and CTC.

## RESULTS

Our study was comprised of 37 patients, including 23 males (62.2%) and 14 females (37.8%). The patients’ age ranged from 33 to 85 years (median: 65 years) at the time of diagnosis. The most cases of gastric tumors are from distal stomach (54.05%) in comparison with tumors arising from the gastric cardia or the gastroesophageal junction. The tumor size varied between 2 and 5 cm (average: 3.5 cm). Seven cases (19%) had advanced tumors at the time of diagnosis.

**Copy number alterations in the CTC and matched tumor tissue**

**Fig. 1:**
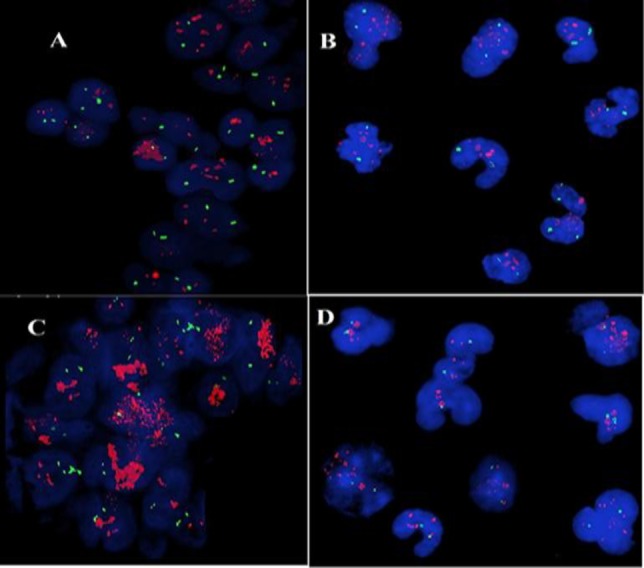
*HER2* and *c-MYC* amplification in paired CTC and tissue samples obtained from GC patients. *HER2* and *c-MYC* amplification in tissue (A and C) and in CTC (B and D), respectively

According to our selected cut-off, *HER2*/CEP17, *MDM2*/CEP12, and *c-MYC*/CEP8 amplification was observed in a total of 2 (5.41%), 1 (2.70%), and 3 (8.11%) out of 37 patients’ tissue samples, respectively (Fig. 1). However, *c-MET* amplification and *TP53* deletion were not observed in our cohort. Overall, amplification of at least one of the selected genes was observed in 3 out of 37 patients. The co-amplification of *HER2*, *MDM2*, and *c-MYC *was observed in one patient, and a simultaneous amplification of *HER2* and *c-MYC* was found in another patient (Table 1). An isolated amplification of the c-*MYC* oncogene was also seen in one of the samples. *HER2*,* MDM2*, and c-*MYC* amplification occurred in the intestinal tumors; however, the copy number alterations of the genes of interest were not detected among the prolonged cancers. By determining the status of *HER2*, *MDM2*, c-*MYC, c-MET*, and *TP53* copy number alteration of the CTC samples obtained from all the patients, we found that *HER2* and *c-MYC* amplification occurred in 1 and 3 out of the 37 patients, respectively. There were no* P53 *or *c-MET* gene copy number alterations in the obtained CTC and tissue samples in our patients. The comparison of the status of *HER2*, *MDM2*, and *c-MYC* genes of the 37 GC patients’ CTC samples with that of the patients’ matched tumor tissues demonstrated that the mean interval between the CTC and tissue samples was equal to 20.1 days. In 36 out of the 37 patients, the *HER2* gene status in the CTC showed a strong harmony with its status in the matched tissue samples (correlation: 97.29%; Kappa: 0.65; *p* < 0.001). *MDM2* amplification was observed in only 1 (2.70%) of the 37 patients’ tissue samples; however, the amplification of this gene was not detectable in the CTC isolated from the patient. *c-MYC *amplification were observed in 3 (8.11%) out of the 37 patients’ samples, and the amplification status in these CTC showed a complete agreement with the status attained from the matched tissue samples (correlation: 100%; Kappa: 1.0, sensitivity: 100%, specificity: 100%.).

**Table 1:** GC samples with gene amplification

**Case** **No.**	**Sex/** **age**	**GC** **types**	**Histological** ** classification**	**Stage**	**Number of CTC analyzed**	***HER2*** ** amplification (% tissue/ %CTC)**	***MDM2*** ** amplification (% tissue/% CTC)**	***c-MYC*** ** amplification (% tissue/% CTC)**
*1*	M/65	intestinal	*Poorly differentiated*	III	100	80/NA	70/NA	90/90
*2*	M/75	intestinal	*Well differentiated*	*II*	*100*	80/80	*NA/NA*	*70/70*
*3*	*M/61*	intestinal	*Adenocarcinoma*	*II*	*100*	*NA/NA*	*NA/NA*	*90/90*

## DISCUSSION

Although recent improvements in the diagnosis and management of GC have resulted in a decrease in overall mortality, most GC patients suffer from advanced or metastatic disease at the time of diagnosis, which is still a major cause of cancer-related deaths in the world^[^^2^^]^. Therefore, development of better diagnostic, prognostic and disease monitoring tools are crucial for the improvement of the clinical outcome of GC patients. Tissue samples were the main sources for evaluating tumor-associated genetic alterations in GC patients. However, the use of tissue samples has several limitations, such as the invasive nature and inability to reflect tumor heterogeneity. Over recent decades, CTC have gained global attention in order to overcome these limitations^[^^12^^]^. Several studies have been indicated the prognostic value of the enumeration of CTC in numerous types of cancers, including GC^[^^13^^,^^14^^]^. Moreover, it has been shown that CTC may represent the genetic compositions of both primary and metastatic tumors, and the real-time assessment of prognostic and therapeutic biomarkers on CTC may be useful to improve the clinical outcome of these patients and also to have a significant effect on targeted cancer therapy^[^^15^^-^^18^^]^. In this study, using the FISH technique, we analyzed the copy number alterations of *HER2*, *MDM2*, *c-MYC*, *c-MET*, and *TP53* in CTC obtained from GC patients to evaluate their application as a potential alternative to tumor biopsy. The importance of *HER2*, *MDM2*, *c-MYC*, and *c-MET* amplification and *TP53* deletion in the progression of GC and their negative effects on the survival of patients with GC have been reported in a number of studies^[^^19^^]^.

In the present study, amplification of *MDM2*, *c-MYC* and *HER2* was shown in a total of three patients who all had intestinal-type GC. The first patient, who showed co-amplification of *MDM2*, *c-MYC*, and *HER2*, was a 65-year-old male. The gastrointestinal endoscopy revealed advanced GC in the cardia of the gastric body, while biopsy results displayed a poor differentiated adenocarcinoma. The clinical diagnosis was T4N2M0, stage III. The second patient, who was a 75-year-old male, indicated co-amplification of *c-MYC* and *HER2*. The gastrointestinal endoscopy showed advanced GC in the cardia of the gastric body, but biopsy results exhibited a well differentiated adenocarcinoma. The clinical diagnosis was T3N0M0, stage II. Finally, the third patient, who showed only *c-MYC* amplification, was a 61-year-old male. The gastrointestinal endoscopy disclosed advanced GC in the antrum of the gastric body; however, the results of biopsy demonstrated a poor differentiated adeno-carcinoma. The clinical diagnosis was T3N0M0, stage II. Although CTC count in patients with GC may act as an early biomarker for the determination of therapeutic efficacy, the prognostic significant of CTC molecular profiling, including determination of *MDM2*, *c-MYC*, and *HER2* status in CTC, is less determined in GC patients. The criteria for assessing biomarker status of *MDM2*, *c-MYC*, and *HER2* in CTC from patients with GC are debatable. However, a recent study has unveiled that CTC molecular profiling in advanced GC patients may improve personalized treatment of patients and may prevent tumor progression^[^^20^^]^. 

As reported in a study, trastuzumab improved the overall survival among patients with *HER2*-positive GC^[^^21^^]^. Therefore, assessing of the *HER2* status in the primary tumor tissue of GC patients is recommended for finding patients who are likely to profit from trastuzumab therapy. The status of *HER2* is mostly determined through analyzing the tumor tissue at the time of initial diagnosis. However, it has been indicated that the *HER2* status may be changed during the progression of disease, and the re-assessment of *HER2* is needed for GC patients who have recurrence or are in the metastatic phase of the disease. Nonetheless, due to the inaccessibility of the tumor tissue, it is often impossible^[^^22^^,^^23^^]^. In this study, we used FISH to evaluate the *HER2* amplification status in the CTC obtained from the GC patients. In our study, *HER2* was amplified in 5.41% of the GC primary tumor tissues. The observed prevalence of *HER2* gene amplification in the primary tumor tissue of GC patients were in the same range, which has been found in a FISH-based study reported in 2002 by Takehana *et al.*^[^^24^^]^ who found *HER2* amplification in approximately 8% of the cases. The *HER2* gene status in the CTC showed a strong concordance with its status obtained from the matched tissue samples in 36 of the 37 patients (97.3%). Among the non-concordant results, only one patient’s tumor tissue showed *HER2* amplification, along with CTC without *HER2* amplification. This difference may be related to the time interval between the time of tissue sampling and CTC *HER2* testing. The results of this study was in agreement with a previous study in which *HER2* concordance of 97% between the CTC and breast cancer tissue samples was reported^[^^25^^]^.

Recent studies have suggested that the overexpression of *c-MYC* may play a role in the tumorigeneses process of GC, and its deregulation is associated with the poor prognostic features of GC patients^[^^26^^,^^27^^]^. Moreover, it has been shown that amplification was the main mechanism of *c-MYC* deregulation in GC^[^^28^^]^. However, c-*MYC* amplification displayed a high degree of heterogeneity in tissue samples obtained from GC patients. It has also been shown that only small parts of a tumor may harbor *c-MYC* amplification^[^^29^^]^. This tumor hetero-geneity decreases the accuracy of diagnosis. In this study, we used FISH to evaluate the *c-MYC* gene amplification status in the CTC and, accordingly, matched tumor tissues obtained from GC patients to evaluate whether CTC can replace tissue sampling for the determination of the *c-MYC* status in GC patients. *c-MYC* amplification was observed in 8.12% of GC cases, and it was the most frequently amplified gene in our study. The observed prevalence of *c-MYC* gene amplification in the primary tumor tissue of GC patients using FISH was within the previously reported range (1.3-7.9%)^[^^30^^,^^31^^]^. The *c-MYC* amplification status of the CTC was in a complete agreement with the status of the matched tissue samples (100%). This high concordance rate suggests that CTC may show a complementary role for tumor tissues in the assessment of the *c-MYC* status in clinical practice, especially for recurrent or metastatic cases. 

In this study, *MDM2* amplification was found in the primary tumor tissue of a single case of GC (2.70%). However, the amplification of this gene was undetectable in the CTC isolated from the same patient. In this case, *MDM2* amplification was seen in 70% of the cells analyzed in the tissue sample. It is possible that with the emergence of new clones without *MDM2* amplification, *MDM2* amplification diminishes during the progression of the disease. 

In this cohort study, we did not observe any *c*-*MET* amplification and *TP53* detention. A previous study has shown that the gain-of-function mutations in *c*-*MET* were exceedingly rare in GC^[32]^. Therefore, due to the small sample size of our study, absence of *c*-*MET* amplification in our patients seems to be rational. 

Our study revealed the feasibility of *HER2* and *c-MYC* status analysis in CTC obtained from GC patients by FISH technique. The concordance between the FISH results of *HER2* and *c-MYC *in CTC and archival tumor tissue showed the potential of using CTC FISH assays for determining the gene status of *HER2* and *c-MYC *in GC patients. Displacement in the *MDM2* and *HER2* status amid archival tumor tissues and CTC (in one case) and also the heterogeneity of *MDM2*, *HER2*, and *c*-*MYC* gene status in the CTC (amplified and non-amplified cells coexisted) suggest that CTC analyses possibly provide important insight about disease heterogeneity, clonal evolution, and clone dynamics.
